# Femoral component failure in the Oxford unicompartmental knee arthroplasty: a case report

**DOI:** 10.1186/1752-1947-8-419

**Published:** 2014-12-11

**Authors:** Kirsten DS Argelo, Mick A Burger, Marco JM Hoozemans, Olivier PP Temmerman

**Affiliations:** 5 CORAL – Centre for Orthopaedic Research Alkmaar, Department of Orthopaedic Surgery, Medical Center Alkmaar, P.O Box 501, 1800 AM Alkmaar, The Netherlands; 6 Research Institute MOVE, Faculty of Human Movement Sciences, VU University Amsterdam, Amsterdam, The Netherlands; 7 Department of Orthopaedic Surgery, Medical Center Alkmaar, P.O Box 501, 1800 AM Alkmaar, The Netherlands

**Keywords:** Complication, Knee arthroplasty, Unicompartmental knee arthroplasty

## Abstract

**Introduction:**

The present case report describes a patient who presented with an early complication after a unicompartmental knee arthroplasty. It is not the first case in this subject but the unique aspect of this case report rests on the timing in which the failure occurred.

**Case presentation:**

A 64-year-old Caucasian man received a medial unicompartmental knee arthroplasty (Oxford® Partial Knee) due to isolated anteromedial osteoarthritis of his right knee. His initial recovery was good, however, after 3 months he presented with acute pain and a locked knee. Radiographs showed a complete loosening and migration of the femoral component. During revision surgery no clear explanation was found for failure of the femoral component.

**Conclusions:**

The most likely explanation for loosening is the combination of peak stresses on the posterior facet of the femoral components of a unicompartmental knee arthroplasty in a patient in a cross-legged knee position causing bone–cement or cement–implant interface failure. Further research is necessary in prosthetic designs and applications of the unicompartmental knee arthroplasty to determine the origin of this early complication.

## Introduction

In orthopaedic patients that present with a painful knee, osteoarthritis is a commonly found diagnosis. The incidence of osteoarthritis of the knee (OAK) in The Netherlands in 2007 was estimated at 1.6:1000 for men and 3.1:1000 for women [1]. End-stage OAK generally results in unicompartmental or total knee arthroplasty. It is expected that the ageing population will increase the need for unicompartmental and total knee arthroplasties [1]. Approximately 10% of the patients that undergo a knee arthroplasty are treated with a unicompartmental knee arthroplasty (UKA) [2]. Specific inclusion criteria are used for UKA, in particular, isolated osteoarthritis in the anteromedial tibiofemoral compartment and an intact anterior cruciate ligament.

For this specific patient population UKA is associated with less invasive surgery and a faster postoperative recovery as compared to total knee arthroplasty [2]. The 10-year survival rate of UKA is reported to be over 90% [3]. However, the absolute number of complications and revisions is expected to increase in accordance with the expected increasing number of UKA that are going to be performed. The present case report describes a Caucasian patient who presented at our orthopaedic outpatient department with an early complication after a UKA.

## Case presentation

**Figure 1:**
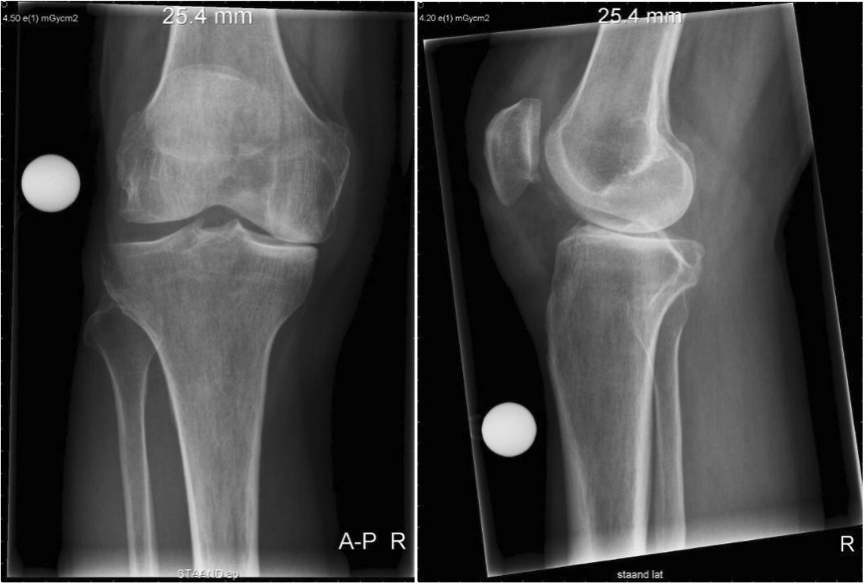
**Preoperative anterior-posterior and lateral X-rays showing anteromedial osteoarthritis of the right knee.**

A 64-year-old Caucasian man visited our out-patient department with symptomatic anteromedial osteoarthritis of his right knee. A physical examination revealed a full range of motion and stable collateral and cruciate ligaments. Plain radiography showed an anteromedial osteoarthritis grade III [4] (Figure 1). After an initial conservative approach, which included a 1-year period of physiotherapy, non-steroidal anti-inflammatory drugs and three hyaluronic acid injections, he was planned for a UKA (Oxford® Partial Knee).

**Figure 2:**
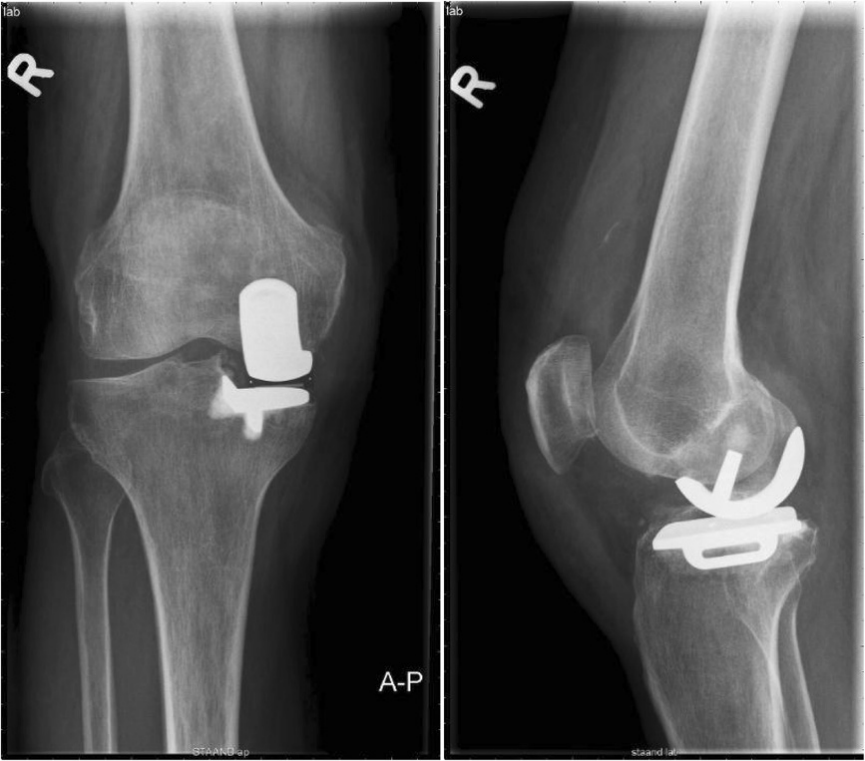
**Postoperative anterior-posterior and lateral X-rays directly after the insertion of an Oxford unicompartmental knee prosthesis.**

Preoperatively, a medium-size femoral component was templated. The surgery was performed by a specialist arthroplasty surgeon. During surgery, a femur of medium size, a tibia size C and an insert size 3 were placed and no complications were experienced. The preoperative diagnosis of anteromedial osteoarthritis was confirmed and the anterior cruciate ligament was found to be stable. The time of blood void was 63 minutes and the bone quality of both tibia and femur was assessed as good. Pulsed lavage was applied to increased femoral cement penetration in combination with the application of retention holes to further enhance cement fixation. Range of motion of the knee after wound closure was 120 degrees of flexion and full extension with stable collateral and cruciate ligaments. The postoperative X-rays revealed no abnormalities and proper positioning of the UKA (Figure 2). The postoperative checks at the out-patient department were planned 6 weeks and 3 months postoperatively. At both appointments the patient showed a good clinical recovery and his range of motion observed at 3 months was 120/0/0.

**Figure 3:**
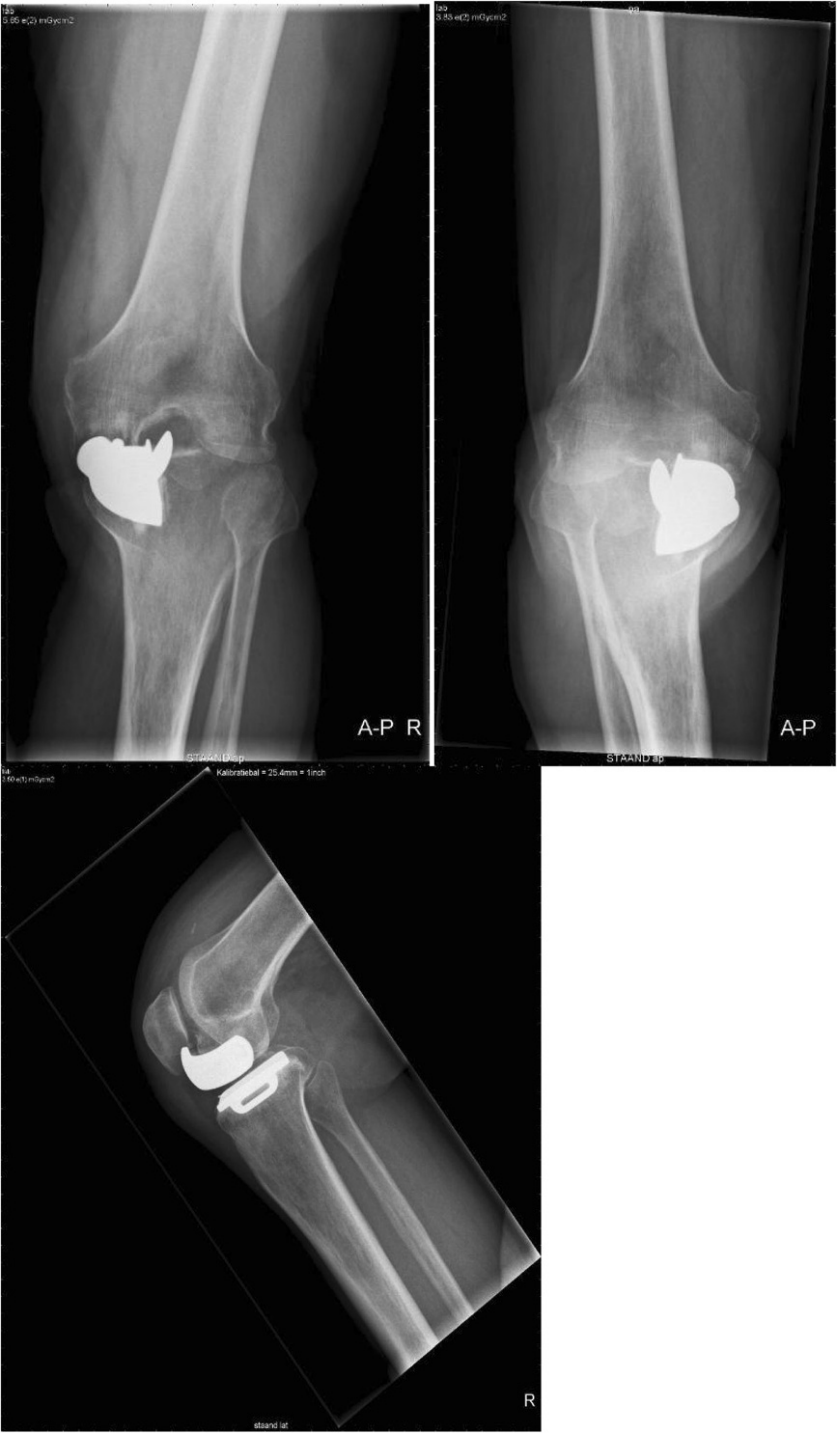
**Anterior-posterior and lateral X-rays at 2 weeks after the 3-month postoperative check-up with the patient reporting extreme pain and a locked knee.**

Two weeks after the last check he presented at the out-patient department with extreme pain and a locked knee. There was no trauma; however, he reported an acute pain after sitting in a cross-legged position. A physical examination showed that he was in pain with a locked knee in 90/90/0. An X-ray showed a fully migrated and loosened femoral component (Figure 3).

**Figure 4:**
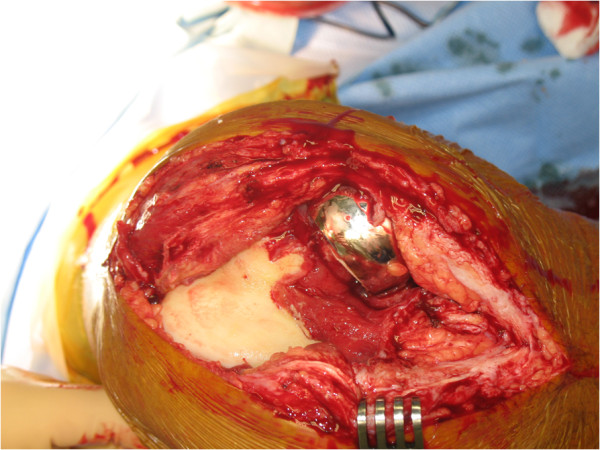
**Photograph of the dislocated femoral component during surgery.**

**Figure 5:**
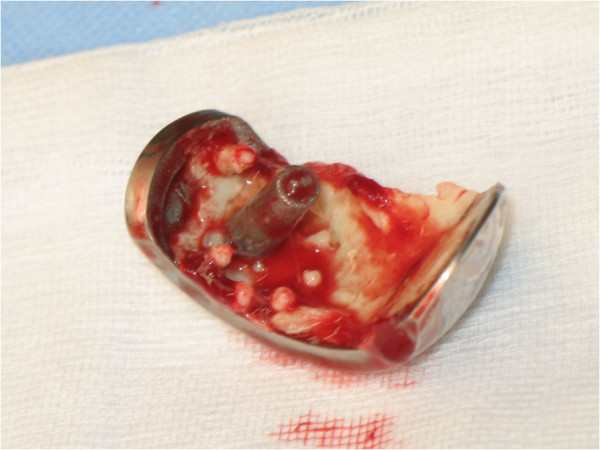
**Photograph of the removed femoral component, with a crack in the cement.**

**Figure 6:**
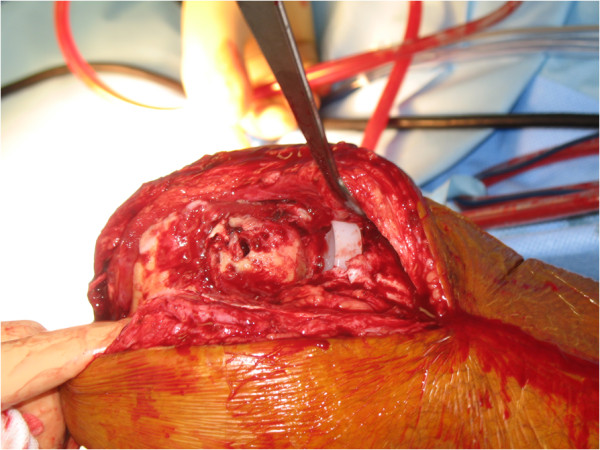
**Photograph of the femoral condyle after removal of the femoral component of the Oxford unicompartmental knee prosthesis.**

During revision surgery, performed by the same surgeon, a crack in the cement of the femoral component was observed (Figures 4, 5 and 6). No other signs were found that could explain failure of the prosthesis. Cultures and biopsies of the medial femur condyle were taken for further examination; however, bacterial infection or osteonecrosis was not found. The tibial component was found to be solid. During revision surgery, a total knee arthroplasty (Genesis II Total Knee System, Smith & Nephew®, Memphis, USA) with a femur size 7 posterior stabilized component was placed in combination with a tibia size 6, an insert size 15 and patella size 26. During surgery no complications were experienced. His range of motion after wound closure was 120 degrees of flexion and full extension with stable collateral ligaments. The postoperative X-rays revealed no abnormalities and a proper positioning of the total knee prosthesis.

His recovery after revision surgery was without complications. During the last appointment at the out-patient department, 1 year after revision surgery, he showed a good clinical recovery and the range of motion observed was 120/0/0.

## Discussion

UKA is a well-established and proven method to reduce symptoms and disabilities associated with (end-stage) anteromedial tibiofemoral osteoarthritis. UKA is less invasive compared to total knee arthroplasty and postoperatively associated with less pain, less blood loss, better proprioception and faster recovery [2, 5–7]. Complications of UKA reported in the literature are aseptic loosening, luxation of the insert, persisting pain postoperatively, persisting stiffness postoperatively, and deep infection [3]. Deep infection is a commonly described complication in arthroplasty [8]. However, in our case, infection was not found in cultures and biopsies. Similarly, other causes and treatments of persistent postoperative anteromedial pain in UKA have been described [2, 9–12]. Concerning failures of the UKA, a loosened tibia component is more often reported [13, 14]. Technical failure, such as making the sagittal cut at the preparation of the tibia too deep, is most often considered the cause of this type of failure.

Although long-term follow-up studies of the Oxford unicompartmental knee prosthesis have shown excellent outcomes, loosening of the femoral component is the second most common cause of revision and the incidence ranges from zero to 2.1% [15]. However, failure of the femoral component in the early postoperative phase is rarely reported in the literature [16]. Monk *et al.*[15] described a technique to detect loosening using lateral radiographs in extension and flexion. If gaps are present between the component and cement on one radiograph and not on the other, the component is considered loose by the authors. We did not acquire these radiographs at the postoperative checks and may therefore have missed loosening of the femoral component.

Another cause of loosening may be smoking. Meldrum *et al*. [17] calculated a 4.5 times greater risk of implant loosening in cigarette smokers. Cigarette smoking has been shown to interfere with bone metabolism, revascularization and bone formation. Our patient smoked for several years 15 to 20 cigarettes a day. However, it is expected that this type of aseptic loosening is seen in a more mid- to long-term follow up and, therefore, is less likely to be an explanation in our case.

Kim *et al.* describe a possible trauma mechanism, which could explain the failure of the femoral component in the present case [18]. They describe a trauma case with an axial vector action in combination with varus and/or valgus stress, which might have caused a fracture of the medial condyle of the femur. Although there was a suspicion for varus stress in our case, deep flexion was observed instead of axial vector action. Therefore, it is unlikely that the failure of the femoral component observed in the present case is caused by axial vector action.

With regard to the operative technique, a pitfall may lie in the posterior femoral saw cut which can be located too far anteriorly. As a result, the posterior part of the femoral component may be inadequately supported, expressed by a wedge sign, which results in an insufficient prosthesis–cement interface [9, 15].

Clarius *et al.* concluded, after a cadaver study in 24 subjects, that the posterior plane facet is proven to be the weak point of the femoral interface because of incompleteness of the cement mantle [9]. In a more recent study Seeger *et al.* further examined the effects of cementing techniques on implant failure in patients with a UKA (Oxford® Partial Knee) [19]. The authors found evidence that the use of pulse lavage decreased the risk of thermal osseous damage and improved cement penetration.

## Conclusions

On reflection, in our case, the cross-legged position may have exerted shear stresses on the posterior plane facet. This, together with an oppressive force on the posterior facet of the femoral component may explain the loosening mechanism in our patient. The visible crack in the cement mantle found during revision surgery may confirm this hypothesis. However, since we have not been able to detect errors in surgical techniques and did apply the current concepts in cementing techniques with regard to preparation and application, the authors feel that future studies should further focus on application and implant design of the UKA.

## Consent

Written informed consent was obtained from the patient for publication of this case report and any accompanying images. A copy of the written consent is available for review by the Editor-in-Chief of this journal.

## Abbreviations

OAK: Osteoarthritis of the knee
UKA: Unicompartmental knee arthroplasty (Oxford® Partial Knee).
